# New York Odontological Society

**Published:** 1892-10

**Authors:** 


### Reports of Society Meetings.

### NEW YORK ODONTOLOGICAL SOCIETY.

A REGULAR meeting of the New York Odontological Society was held on Tuesday evening, June 21, 1892, at the New York Academy of Medicine, the Vice-President, Dr. Brockway, being in the chair.

There being no incidents of office practice or casual communications, the chairman announced the two papers which were to be read,—namely, "A Contribution to the Minute Anatomy of the Cementum," by Prof. Carl Heitzmann and F. A. Roy, M.D., D.D.S., of New York, and "Partial Suppurating Pulpitis—Origin of Certain Alveolar Abscesses in Living Teeth," by Dr. A. C. Hugenschmidt, of Paris, France.

(For the above-mentioned papers, see pages 709 and 741, respectively).

*Dr. Littig.*—I notice that there are very few gentlemen present who understand the first paper on the list. There would probably be no discussion of it, and I think we had better have the papers read by title only, and published in the Transactions, and we can read them at our leisure. They can be printed as our usual proceedings, without any discussion.

*Dr. Heitzmann.*—I am perfectly willing to read the paper. I have to offer a number of drawings; they are few in number, but perhaps the quality will make up for the quantity.

*Dr. Howe.*—On the subject of the motion, I do not see any objection to it whatever, unless there is some one present who wishes to discuss the subject. I would rather favor the idea of passing the papers without reading them, if it meet the approval of those present.

*Dr. Lord.*—It would seem very desirable to hear what Dr. Heitzmann has to say on the subject of the paper. He has undoubtedly read it, and he should give us his views, which may be lost entirely to us if we do not get them this evening, to be published with the paper, and if there are others who wish to discuss the paper, as has been remarked, it would be desirable to have it read.

*Dr. Heitzmann.*—I have to remark that a slight alteration was made in the title of the paper. Dr. Roy is engaged in practice in New Orleans, and I therefore had a little more work in getting up the text than usual; consequently my name appears with Dr. Roy's on the paper. I have therefore no views to announce different from those in the paper. What will be printed is exactly what I mean on the topic.

A motion was then made that the papers be read by title and passed, which was carried.

*Dr. Remington.*—I would suggest that if any one present has anything to say on the subject of the second paper, that he be invited to do so.

As none of the gentlemen present desired to speak on the paper, the subject was passed.

Adjourned.

